# Supplementary material for: Distinctive mechanisms of epilepsy-causing mutants discovered by measuring S4 movement in KCNQ2 channels
Source: eLife. 2022 Jun 1;11:e77030. doi: 10.7554/eLife.77030 (PMC9197397; doi:10.7554/eLife.77030)
Supplement: Supplementary file 1. — Data are mean ± SEM, n=number of cells. [file elife-77030-supp1.docx]

**Supplementary File 1.** **Biophysical properties of wild type and mutant KCNQ2 channels. V_1/2_ and F_1/2_ of activation; V_1/2_ of state dependent MTS modification, and the second order rate constant of KCNQ2 channels. Data are mean ± SEM, n = number of cells.**

.

| Constructs | n | V_1/2_ (mV) | n | F_1/2_ (mV) | n | V_1/2_ (mV) open state MTSET | n | V_1/2_ (mV) closed state MTSET | Second order rate constant (M^−1^s^−1^)  (open state) | Second order rate constant  (M^−1^s^−1^) (closed state) |
| --- | --- | --- | --- | --- | --- | --- | --- | --- | --- | --- |
| wt-KCNQ2 (+MTSET) | 21 | −43 ± 0.7 |  | - | 5 | −42.1 ± 0.5 | 3 | −44.2 ± 0.5 | - | - |
| Q188C | 7 | −33.7 ± 0.3 | - | - | - | - | - | - | - | - |
| G189C | 8 | -18.8 ± 0.5 | - | - |  |  |  |  |  |  |
| N190C Closed/Open | 24 | −13.2 ± 0.8 | - | - | 9 | −20.2 ± 1.7 | 11 | −19.5 ± 1.3 | - | - |
| N190C  ONLY Open | 24 | -13.2 ± 0.8 | - | - | 5 | −24.8 ± 9.7 |  |  | - | - |
| V191C | 5 | −45.0 ± 1.2 | - | - | - | - | - | - | - | - |
| F192C | 9 | −55.8 ± 0.8 | - | - | - | - | - | - | - | - |
| A193C | 12 | −70 ± 2.4 | - |  | 7 | −111.7 ± 1.8 | 3 | −65.8 ± 3.4 | 11800 ± 2.6  at +20-mV | 2080.3 ± 1.9  at −100-mV |
| S195C | 5 | −28.3 ± 3.2 | - | - | 3 | −83.5 ± 4.0 | 3 | −29.5 ± 2.8 | 11350 ± 0.01 | 38.6 ± 1.1 |
| A196C | 15 | −30.6 ± 0.7 | - | - | 3 | −57.5 ± 11.4 | 3 | −30.8 ± 0.7 | 1400 ± 1.1 | 94.3 ± 9.6 |
| R198C | 13 | −30.4 ± 1.0 | - | - | 8 | −90.2 ± 1.4 | 8 | −31.7 ± 1.8 | 3230 ± 2.05 | 7.03 ± 3.2 |
| S199C | 10 | −49.6 ± 0.8 | - | - | 3 | −49.5 ± 0.6 | 3 | −47.5 ± 1.2 | 334.6 ± 3.2 | 11.9 ± 1.7 |
| L200C | 3 | −31.9 ± 0.3 | - | - | 3 | −41.2 ± 1.4 | 3 | −31.9 ± 0.3 | 28.2 ± 5.8 | 4.4 ± 7.9 |
| R201C | 4 | Voltage independent | - | - | - | - | - | - | - | - |
| F202C | 4 | −31.4 ± 2.6 | - | - | 3 | −31 ± 2.6 | 3 | −31.3 ± 2.3 | - | - |
| F192C/Alexa488 maleimide (KCNQ2*) | 9 | −77.1 ± 2.7 | 8 | −87.1 ± 3.9 | - | - | - | - | - | - |
| F192C/  Dylight-488 maleimide | 11 | −79.9 ± 1.4 | 8 | −94.7 ± 1.8 | - | - | - | - | - | - |
| KCNQ2*-R198Q | 10 | −110.3 ± 3.5 | 4 | −119.8 ± 4.2 | - | - | - | - | - | - |
| KCNQ2*-R214W | 8 | −17.1 ± 0.9 | 7 | −77 ± 0.6 | - | - | - | - | - | - |
